# Supplementary material for: Triel Bond Formed by Malondialdehyde and Its Influence on the Intramolecular H-Bond and Proton Transfer
Source: Molecules. 2022 Sep 18;27(18):6091. doi: 10.3390/molecules27186091 (PMC9505241; doi:10.3390/molecules27186091)
Supplement: Supplementary file 1 [file molecules-27-06091-s001.zip › molecules-1911996-supplementary.pdf]

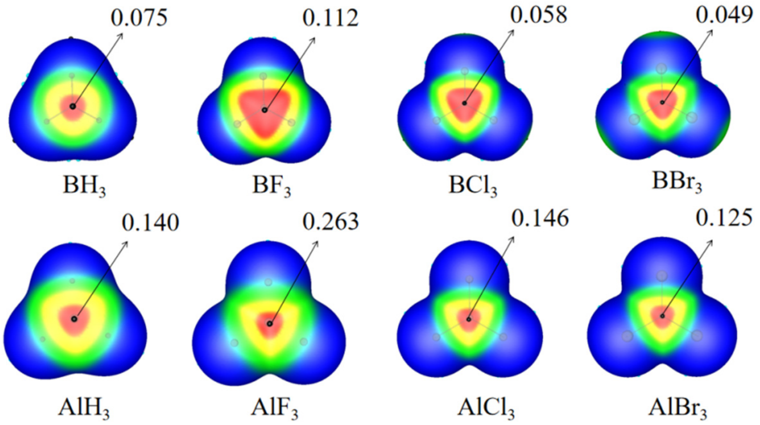

Figure S1. MEP maps of TrX_3. Color ranges are: red, greater than 0.02; yellow, between 0.02 and 0; green, between -0.02 and 0; blue, less than -0.02. All are in a.u.

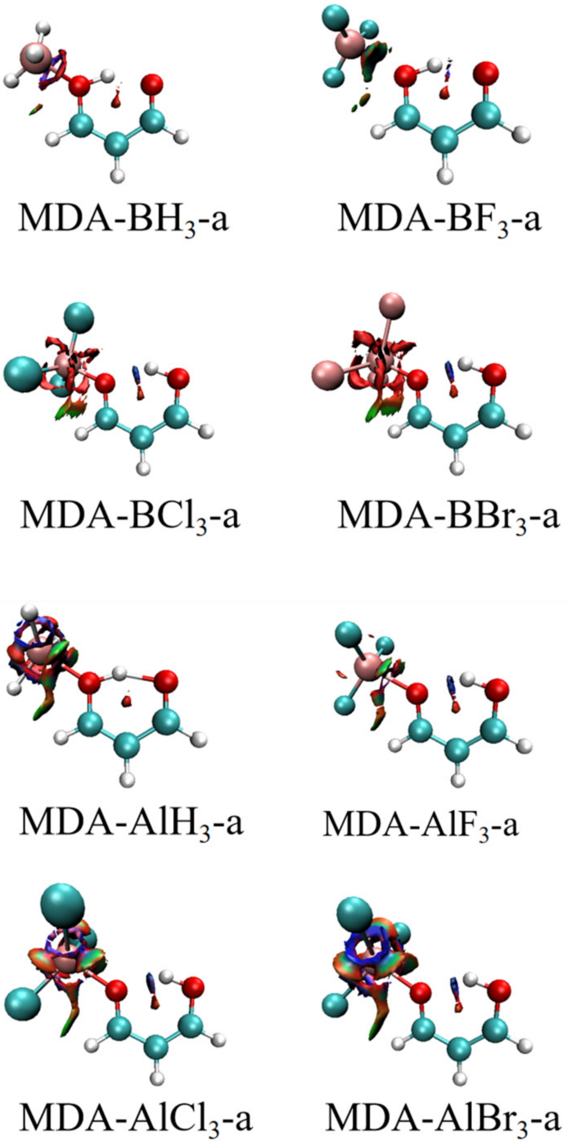

Figure S2. NCI diagram of binary complex formed by the hydroxyl O with TrX_3. Blue, green, and red areas represent strong attraction, weak attraction, and strong repulsion, respectively. Diagrams are drawn by the Multiwfn and the VMD programs

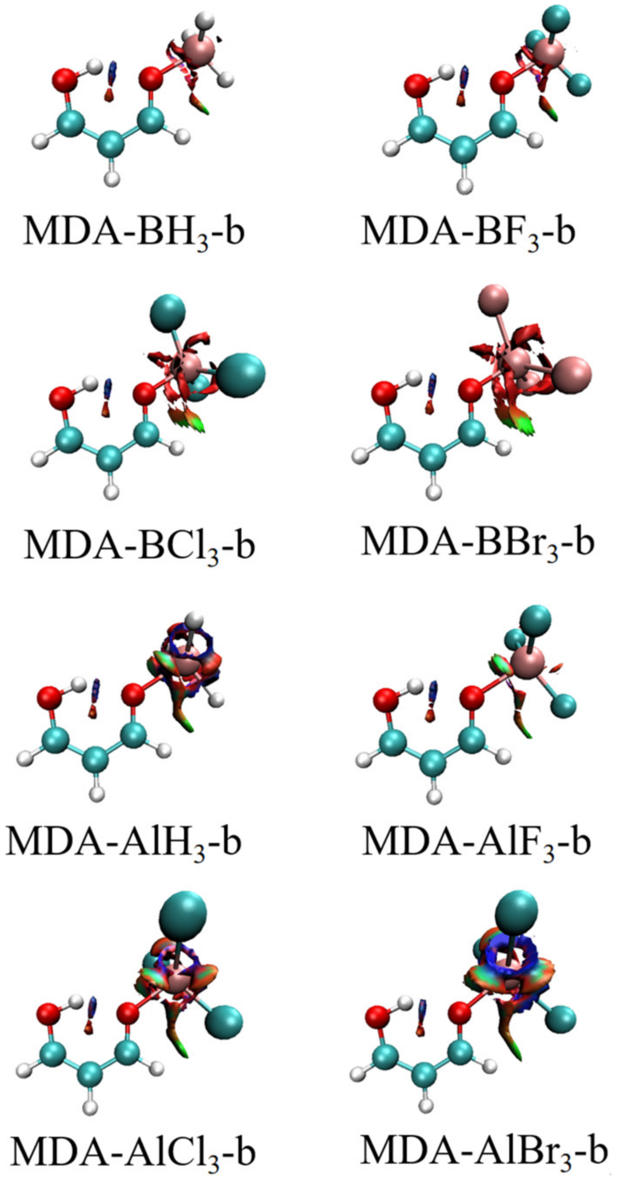

Figure S3. NCI diagram of binary complex of MDA formed by the carbonyl O with TrX_3. Blue, green, and red areas represent strong attraction, weak attraction, and strong repulsion, respectively.

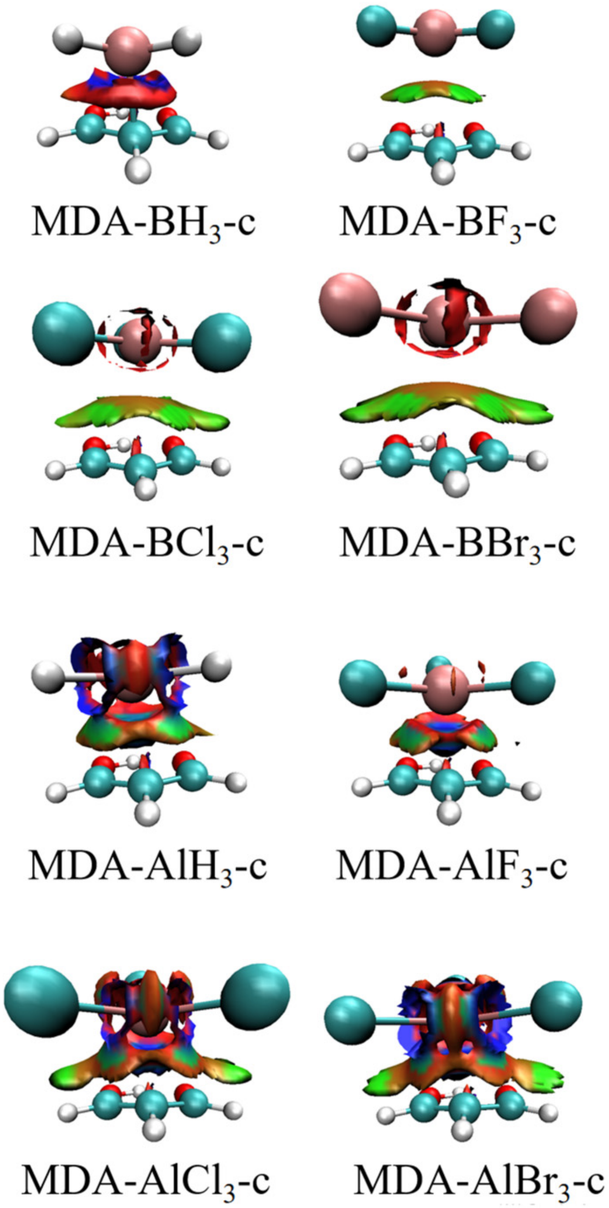

Figure S4. NCI diagram of π-π structures formed by the carbon center of MDA with TrX_3. Blue, green, and red areas represent strong attraction, weak attraction, and strong repulsion, respectively.

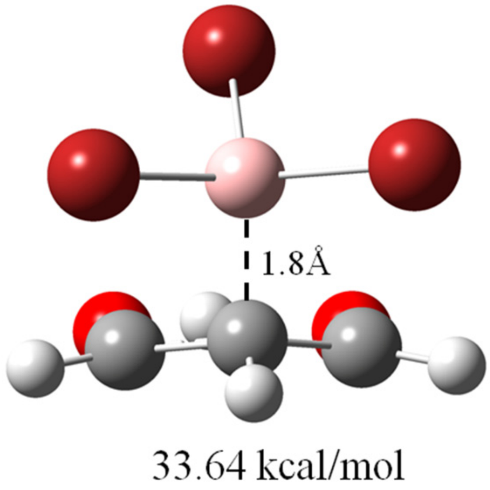

Figure S5. Structure of MDA-BBr_3-c at a B···C distance of 1.8 Å.

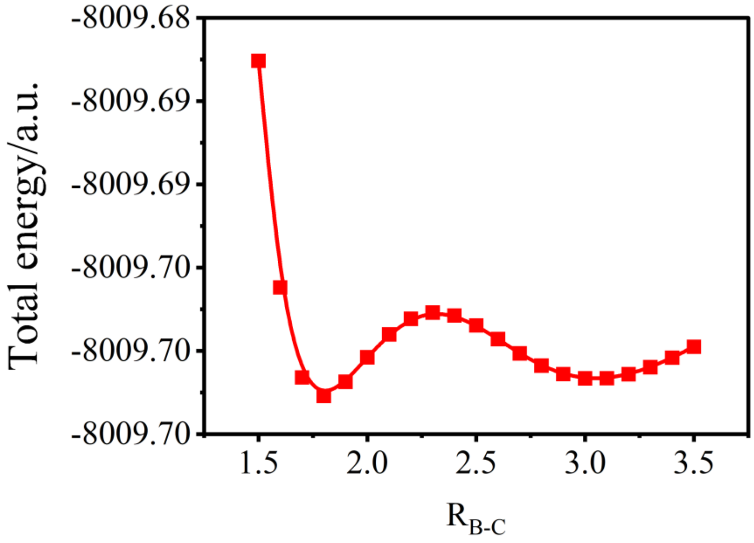

Figure S6. The energy curve of MDA-BBr_3-c by changing the B···C distance from 1.5 to 3.5 Å

Table S1. Charge transfer (CT, e) in the complexes

	CT
MDA-BH_3-a	0.211
MDA-BF_3-a	0.040
MDA-BCl_3-a	0.294
MDA-BBr_3-a	0.286
MDA-AlH_3-a	0.101
MDA-AlF_3-a	0.108
MDA-$AlCl_3$-a	0.131
MDA-$AlBr_3$-a	0.127
MDA-BH_3-b	0.250
MDA-BF_3-b	0.220
MDA-BCl_3-b	0.294
MDA-BBr_3-b	0.286
MDA-AlH_3-b	0.115
MDA-AlF_3-b	0.108
MDA-$AlCl_3$-b	0.131
MDA-$AlBr_3$-b	0.127
MDA-BH_3-c	0.268
MDA-BF_3-c	0.010
MDA-BCl_3-c	0.015
MDA-BBr_3-c	0.022
MDA-AlH_3-c	0.112
MDA-AlF_3-c	0.120
MDA-$AlCl_3$-c	0.160
MDA-$AlBr_3$-c	0.162
BH_2-MDA-BH_3-a	0.244
BH_2-MDA-BF_3-a	0.022
BH_2-MDA-AlH_3-a	0.112

Note: CT is calculated as a sum of NBO charge on the each atom in TrX_3

Table S2. Electrostatic (E^{ele}), exchange (E^{ex}), repulsion (E^{rep}), polarization (E^{pol}), and dispersion energies (E^{disp}) as well as the total interaction energy (ΔE^{total}) of triel bond in the binary complexes. All are in kcal/mol.

	E^{ele}	E^{ex}	E^{rep}	E^{pol}	E^{disp}	ΔE^{total}
MDA-BH_3-a	-44.69	-75.99	146.59	-37.48	-8.52	-20.09
MDA-BF_3-a	-17.34	-20.88	39.94	-7.64	-1.64	-7.56
MDA-BCl_3-a	-92.89	-142.70	287.76	-87.74	-2.07	-37.64
MDA-BBr_3-a	-102.76	-163.77	329.93	-99.07	-2.52	-38.19
MDA-AlH_3-a	-38.42	-41.82	81.68	-20.46	-2.37	-21.39
MDA-AlF_3-a	-59.70	-36.82	80.60	-32.93	6.19	-42.65
MDA-$AlCl_3$-a	-65.42	-60.15	121.92	-40.16	1.80	-42.01
MDA-$AlBr_3$-a	-68.46	-70.01	139.90	-43.03	1.02	-40.58
MDA-BH_3-b	-57.90	-91.72	177.72	-49.43	-7.52	-28.86
MDA-BF_3-b	-63.33	-76.70	157.53	-50.10	3.09	-29.50
MDA-BCl_3-b	-92.89	-142.70	287.76	-87.74	-2.07	-37.64
MDA-BBr_3-b	-102.76	-163.77	329.93	-99.07	-2.52	-38.19
MDA-AlH_3-b	-48.59	-48.16	94.71	-25.40	0.02	-27.42
MDA-AlF_3-b	-59.70	-36.82	80.60	-32.93	6.19	-42.65
MDA-$AlCl_3$-b	-65.42	-60.15	121.92	-40.16	1.80	-42.01
MDA-$AlBr_3$-b	-68.46	-70.01	139.90	-43.03	1.02	-40.58
MDA-BH_3-c	-39.97	-91.42	169.88	-43.18	-13.80	-18.49
MDA-BF_3-c	-5.71	-10.74	19.86	-2.32	-3.92	-2.82
MDA-BCl_3-c	-5.57	-16.31	27.88	-1.99	-8.23	-4.22
MDA-BBr_3-c	-7.74	-23.58	40.29	-2.89	-10.51	-4.42
MDA-AlH_3-c	-22.86	-40.61	75.82	-17.75	-6.96	-12.36
MDA-AlF_3-c	-30.26	-35.29	74.60	-28.88	-2.41	-22.24
MDA-$AlCl_3$-c	-38.09	-60.47	118.27	-35.22	-8.83	-24.34
MDA-$AlBr_3$-c	-42.10	-71.83	138.66	-37.95	-10.53	-23.74

MDA

C	1.17962100	0.43638800	-0.00020600
O	1.28458700	-0.88012700	-0.00008800
H	0.34130700	-1.21644300	0.00031300
C	-0.00981600	1.10190500	0.00002200
C	-1.23353400	0.35282400	0.00034600
H	-2.17877100	0.91125300	0.00027700
O	-1.27085300	-0.89114500	-0.00003000
H	2.13401000	0.94903900	-0.00053800
H	-0.02404100	2.17962300	-0.00007300

BH_3

B	0.00000000	0.00000000	0.00000000
H	0.00000000	1.18725200	0.00000000
H	1.02819000	-0.59362600	0.00000000
H	-1.02819000	-0.59362600	0.00000000

BF_3

B	0.00000000	0.00000000	0.00000000
F	0.00000000	1.31702700	0.00000000
F	-1.14057900	-0.65851400	0.00000000
F	1.14057900	-0.65851400	0.00000000

BCl_3

B	0.00000000	0.00000000	0.00000000
Cl	0.00000000	1.73990400	0.00000000
Cl	-1.50680100	-0.86995200	0.00000000
Cl	1.50680100	-0.86995200	0.00000000

BBr_3

B	0.00000000	0.00000000	0.00000000
Br	0.00000000	1.88918600	0.00000000
Br	-1.63608300	-0.94459300	0.00000000
Br	1.63608300	-0.94459300	0.00000000

AlH_3

Al	0.00000000	0.00000000	0.00000000
H	0.00000000	1.57987100	0.00000000
H	-1.36820900	-0.78993600	0.00000000
H	1.36820900	-0.78993600	0.00000000

AlF_3

Al	0.00000000	0.00000000	0.00000000
F	0.00000000	1.64708700	0.00000000

F	1.42641900	-0.82354300	0.00000000
F	-1.42641900	-0.82354300	0.00000000

$AlCl_3$

Al	0.00000000	0.00000000	0.00000000
Cl	0.00000000	2.08148000	0.00000000
Cl	-1.80261500	-1.04074000	0.00000000
Cl	1.80261500	-1.04074000	0.00000000

$AlBr_3$

Al	0.00000000	0.00000000	0.00000000
Br	0.00000000	2.23184500	0.00000000
Br	-1.93283500	-1.11592300	0.00000000
Br	1.93283500	-1.11592300	0.00000000

MDA-BH_3-a

C	0.30351200	1.01267600	-0.10836400
O	0.92839500	-0.15878800	-0.29534200
H	0.21834100	-0.88887600	-0.19792600
C	-1.03768200	1.06825100	0.06504300
C	-1.79060500	-0.16101600	0.10944200
H	-2.87027700	-0.10599900	0.28521600
O	-1.26153100	-1.27711500	-0.03363800
H	0.97384600	1.86107800	-0.11693400
H	-1.52512400	2.02065800	0.19334300
B	2.54254800	-0.35748200	0.23261100
H	3.03122900	0.65471400	-0.19273800
H	2.42322200	-0.41588100	1.42534600
H	2.84976800	-1.37053200	-0.32424600

MDA-BF_3-a

C	-0.94072600	1.04158300	-0.04603800
O	-0.21083900	-0.06873800	-0.10633000
H	-0.87383800	-0.83440300	-0.08113500
C	-2.29736400	1.01656500	0.02241400
C	-2.97287200	-0.25232500	0.03663800
H	-4.06761300	-0.25833800	0.09533400
O	-2.36829400	-1.33901500	-0.01399700
H	-0.35331000	1.95022300	-0.05984700
H	-2.85207500	1.93928900	0.06729200
B	1.97480700	-0.08071000	0.02194000
F	2.04928700	1.22561500	-0.22210100
F	2.15259000	-0.93923600	-0.96924200
F	2.03941500	-0.50483900	1.27503100

MDA-BCl_3-a

C	1.20789900	-1.05049600	-0.00057600
O	0.58055300	0.05992600	-0.00018300
H	1.85239100	1.24125800	-0.00033200
C	2.60634200	-1.07134500	-0.00012100
C	3.33395500	0.09766500	-0.00005300
H	4.41717300	0.08460300	-0.00014000
O	2.83865100	1.30420700	-0.00020400
H	0.62095200	-1.96668100	-0.00128900
H	3.12685500	-2.01415500	0.00009700
B	-1.00822100	0.07039900	0.00004500
Cl	-1.50799200	-0.81390700	-1.51789300
Cl	-1.40977000	1.83534200	-0.00016300
Cl	-1.50688900	-0.81349600	1.51858800

MDA-BBr_3-a

C	-3.88516900	-0.02720600	0.00004200
O	-3.39728100	-1.23486700	-0.00121700
H	-2.40976500	-1.18077500	-0.00130900
C	-3.15241300	1.14091400	0.00109100
C	-1.75711800	1.11507200	0.00087600
H	-1.15968700	2.02410600	0.00165500
O	-1.13943400	-0.00463000	-0.00058900
H	-4.96837700	-0.00826200	0.00033000
H	-3.67021200	2.08522100	0.00220100
B	0.42606000	-0.04535200	-0.00038300
Br	1.00454200	0.89848800	-1.64463000
Br	1.00449000	0.89696400	1.64518400
Br	0.82352900	-1.97117300	-0.00051300

MDA-AlH_3-a

C	-0.27619700	1.06526800	0.00007700
O	0.44238200	-0.06663700	0.00007100
H	-0.25614400	-0.83697900	0.00010300
C	-1.63127100	1.02371300	-0.00008400
C	-2.28387900	-0.25770900	-0.00005500
H	-3.37739200	-0.29603700	-0.00008900
O	-1.64773300	-1.32996700	0.00003300
H	0.32602000	1.96406800	0.00029000
H	-2.20026800	1.93841800	-0.00007600
Al	2.47816700	-0.16398200	-0.00003700
H	2.72150100	-0.94022900	1.36593200
H	2.72153100	-0.94029000	-1.36598000

H	2.63948000	1.42801600	-0.00016800

MDA-AlF$_3$-a

C	-1.00603300	1.03161100	-0.00077600
O	-0.33434200	-0.04814000	-0.00027600
H	-1.59247400	-1.23662200	0.00004500
C	-2.41046300	1.02452900	0.00022800
C	-3.11012900	-0.15779600	-0.00020600
H	-4.19310500	-0.17705000	-0.00062300
O	-2.57452100	-1.34998800	-0.00008000
H	-0.45242900	1.97018700	-0.00171700
H	-2.94929100	1.95698000	0.00030400
Al	1.57921700	-0.07868700	-0.00009800
F	1.84447100	1.58116600	0.00056500
F	1.91709000	-0.88485200	-1.42112800
F	1.91489700	-0.88471400	1.42174700

MDA-AlCl$_3$-a

C	-1.00603300	1.03161100	-0.00077600
O	-0.33434200	-0.04814000	-0.00027600
H	-1.59247400	-1.23662200	0.00004500
C	-2.41046300	1.02452900	0.00022800
C	-3.11012900	-0.15779600	-0.00020600
H	-4.19310500	-0.17705000	-0.00062300
O	-2.57452100	-1.34998800	-0.00008000
H	-0.45242900	1.97018700	-0.00171700
H	-2.94929100	1.95698000	0.00030400
Al	1.57921700	-0.07868700	-0.00009800
F	1.84447100	1.58116600	0.00056500
F	1.91709000	-0.88485200	-1.42112800
F	1.91489700	-0.88471400	1.42174700

MDA-AlBr$_3$-a

C	-2.01667000	1.26686700	0.00194500
O	-1.35443700	0.17915000	0.00078000
H	-2.62387300	-1.01330000	-0.00076000
C	-3.41799300	1.26665900	0.00190700
C	-4.12787900	0.08799700	0.00049400
H	-5.21113500	0.08059100	0.00039800
O	-3.60735800	-1.10871400	-0.00089300
H	-1.45245300	2.19976800	0.00291300
H	-3.95184600	2.20214500	0.00294000
Al	0.53404900	-0.05401200	0.00008900
Br	0.83685800	-1.21364300	1.92629400

Br	0.83602200	-1.20753300	-1.92997400
Br	1.28044200	2.10518600	0.00277100

MDA-BH_3-b

C	-1.81382500	-0.00939700	0.00017500
O	-1.41146300	-1.26100800	-0.00001500
H	-0.42369100	-1.25416600	-0.00033700
C	-0.99054500	1.08230300	0.00008100
C	0.41757700	0.93254600	-0.00022700
H	1.07666200	1.80144000	-0.00013100
O	0.95839300	-0.20410100	-0.00058700
H	-2.89206700	0.09025600	0.00063500
H	-1.42204900	2.06930800	0.00037100
B	2.60035400	-0.36720700	0.00043100
H	2.99298300	0.77507900	0.00184200
H	2.80673200	-0.97791700	-1.01312600
H	2.80497500	-0.97980000	1.01323300

MDA-BF_3-b

C	2.82289900	-0.10655500	0.00230100
O	2.31988000	-1.31643000	-0.00099400
H	1.33559100	-1.23244500	-0.00445800
C	2.09429700	1.05438700	0.00075800
C	0.68460400	1.02480700	-0.00414700
H	0.10423400	1.94576700	-0.00699900
O	0.05017700	-0.06838900	-0.00686100
H	3.90587100	-0.09678500	0.00589000
H	2.60901100	2.00041400	0.00295200
B	-1.64403600	-0.10207000	0.00090000
F	-1.91170700	-0.84319600	-1.10198900
F	-1.99222300	1.21772000	-0.08342000
F	-1.90782400	-0.69273300	1.19290700

MDA-BCl_3-b

C	3.33404900	-0.09773700	0.00000300
O	2.83870900	-1.30431800	0.00007500
H	1.85243500	-1.24102600	0.00007300
C	2.60640300	1.07124300	-0.00001000
C	1.20793700	1.05042400	0.00000600
H	0.62105100	1.96664000	-0.00002100
O	0.58057800	-0.05997400	0.00007500
H	4.41726400	-0.08468400	0.00000800
H	3.12686700	2.01408700	-0.00004300
B	-1.00819500	-0.07028300	0.00000800

Cl	-1.50730000	0.81369600	-1.51828700
Cl	-1.41003200	-1.83528400	-0.00000100
Cl	-1.50744900	0.81377400	1.51821600

MDA-BBr_3-b

C	-3.88516900	-0.02720600	0.00004200
O	-3.39728100	-1.23486700	-0.00121700
H	-2.40976500	-1.18077500	-0.00130900
C	-3.15241300	1.14091400	0.00109100
C	-1.75711800	1.11507200	0.00087600
H	-1.15968700	2.02410600	0.00165500
O	-1.13943400	-0.00463000	-0.00058900
H	-4.96837700	-0.00826200	0.00033000
H	-3.67021200	2.08522100	0.00220100
B	0.42606000	-0.04535200	-0.00038300
Br	1.00454200	0.89848800	-1.64463000
Br	1.00449000	0.89696400	1.64518400
Br	0.82352900	-1.97117300	-0.00051300

MDA-AlH_3-b

C	2.29990900	-0.10717900	0.00001600
O	1.79961900	-1.31919400	-0.00005600
H	0.81415500	-1.22752700	-0.00021400
C	1.56132800	1.04692200	0.00011000
C	0.14999900	0.99945000	-0.00003900
H	-0.43434300	1.92194600	-0.00015900
O	-0.48296200	-0.09428100	-0.00015000
H	3.38289000	-0.09203800	0.00021000
H	2.06583000	1.99862700	0.00027000
Al	-2.48115100	-0.18478700	0.00011900
H	-2.72948300	-0.96571600	1.36633900
H	-2.72967200	-0.96409100	-1.36701600
H	-2.71508200	1.40366200	0.00015800

MDA-AlF_3-b

C	3.11082100	-0.15730700	-0.00029300
O	2.57597200	-1.34975500	-0.00062500
H	1.59400200	-1.23675700	-0.00029100
C	2.41048800	1.02458800	0.00023600
C	1.00592100	1.03117900	0.00065600
H	0.45231500	1.96976000	0.00079100
O	0.33439600	-0.04853700	0.00047100
H	4.19382000	-0.17589900	-0.00087500
H	2.94882200	1.95732200	0.00023300

Al	-1.57975400	-0.07887000	0.00006700
F	-1.91610600	-0.88493600	1.42154600
F	-1.91676700	-0.88419400	-1.42152700
F	-1.84473600	1.58095900	-0.00036400

MDA-$AlCl_3$-b

C	3.59549600	-0.13916000	0.00007300
O	3.02557900	-1.31357600	0.00016600
H	2.04729000	-1.17620000	0.00017000
C	2.93428800	1.06688700	0.00002800
C	1.53327200	1.12468700	0.00003300
H	1.01286900	2.08244400	-0.00009700
O	0.82494900	0.06718300	0.00015300
H	4.67750100	-0.19122800	0.00004400
H	3.50532400	1.98012300	-0.00005300
Al	-1.07674900	-0.06182300	0.00000500
Cl	-1.42515400	-1.12244200	-1.79772200
Cl	-1.64477500	1.99213700	-0.00214400
Cl	-1.42582500	-1.11879900	1.79966200

MDA-$AlBr_3$-b

C	-4.12800900	-0.08798800	-0.00201000
O	-3.60756500	1.10876300	-0.00189000
H	-2.62408200	1.01340900	-0.00155200
C	-3.41803100	-1.26659700	-0.00171200
C	-2.01670100	-1.26676400	-0.00119400
H	-1.45252200	-2.19969200	-0.00081300
O	-1.35445900	-0.17905500	-0.00112500
H	-5.21126300	-0.08066800	-0.00234800
H	-3.95179000	-2.20214100	-0.00180100
Al	0.53407400	0.05405600	0.00014700
Br	0.83451800	1.21143800	1.92813300
Br	0.83848900	1.20961800	-1.92824300
Br	1.28040300	-2.10514800	0.00177300

MDA-BH_3-c

C	-0.88405700	-0.70467600	0.47256000
O	-0.35519400	-1.63517600	-0.26664000
H	0.55539300	-1.29398800	-0.52037100
C	-0.26852700	0.50416200	0.74762700
C	1.11074700	0.69197400	0.32334600
H	1.61093900	1.61903700	0.62840500
O	1.71220200	-0.13296500	-0.37177600
H	-1.87180000	-0.93921800	0.85038100

H	-0.68435100	1.13443400	1.51800000
B	-1.24022400	1.29164400	-0.76949200
H	-2.39636300	1.14171200	-0.48846200
H	-0.80904400	0.67804700	-1.70093500
H	-0.80869700	2.39812800	-0.59342900

MDA-BF_3-c

C	1.13486500	-1.18310800	0.59923400
O	1.80433600	-1.36920600	-0.51933000
H	2.03727200	-0.45017200	-0.84342500
C	0.85705900	0.04737100	1.12300800
C	1.34255500	1.22407400	0.45197600
H	1.12867800	2.20001700	0.90576700
O	1.98450000	1.17856000	-0.61120300
H	0.81149700	-2.10208300	1.07385400
H	0.32237800	0.12569600	2.05645700
B	-1.68241700	0.03590100	-0.21538200
F	-2.11669500	-1.02204200	0.44481100
F	-1.88836900	1.24397900	0.27748600
F	-1.12885800	-0.10613800	-1.40193900

MDA-BCl_3-c

C	-1.69367400	-1.18705000	-0.79370700
O	-2.39908200	-1.37270000	0.30469300
H	-2.61754300	-0.45115600	0.63314500
C	-1.37445900	0.04338200	-1.29103900
C	-1.84213200	1.21946900	-0.61139400
H	-1.58990200	2.19711000	-1.04190400
O	-2.51153500	1.17543500	0.43634900
H	-1.37896000	-2.10693000	-1.27231700
H	-0.80099700	0.12342800	-2.20093500
B	1.31450800	0.02094100	0.24401100
Cl	1.90021600	-1.40217600	-0.57930000
Cl	1.64496800	1.59965000	-0.42521600
Cl	0.48784000	-0.12358200	1.76395500

MDA-BBr_3-c

C	-1.96777800	-1.19484200	-1.28416000
O	-2.88029600	-1.41419300	-0.35848200
H	-3.16758800	-0.50262300	-0.05517200
C	-1.56275100	0.05065100	-1.66929400
C	-2.17258500	1.20473800	-1.07000700
H	-1.85026700	2.19477600	-1.41667900
O	-3.03892100	1.12782100	-0.18016400

H	-1.55377400	-2.09906300	-1.71431600
H	-0.81726600	0.15868800	-2.44104400
B	0.77016100	0.01478500	0.28921400
Br	-0.38805500	-0.19678500	1.75918900
Br	1.57838700	-1.49622100	-0.52148200
Br	1.24139700	1.75306400	-0.30539100

MDA-AlH_3-c

C	-0.45663400	1.18208300	0.50863500
O	-1.28654600	1.38448300	-0.47722200
H	-1.57555000	0.47046800	-0.78028900
C	-0.08649200	-0.06807500	0.95323000
C	-0.74259900	-1.23355000	0.38997000
H	-0.50926300	-2.21184900	0.82500800
O	-1.54015200	-1.15222000	-0.55373800
H	-0.06134300	2.08898600	0.95238400
H	0.50518500	-0.15337500	1.85373100
Al	1.97418600	-0.08057500	-0.34410200
H	1.32772700	0.19179000	-1.76708400
H	2.70713100	1.10124800	0.42750400
H	2.26963100	-1.58064200	0.09873800

MDA-AlF_3-c

C	1.12069200	-1.18960600	0.60799700
O	2.00788700	-1.35406800	-0.31935600
H	2.28101300	-0.42342200	-0.60754000
C	0.65850300	0.05417200	1.01068500
C	1.33883200	1.23891200	0.50021300
H	1.06040300	2.21058800	0.92055700
O	2.19954600	1.16806500	-0.38449600
H	0.73707400	-2.10907600	1.03723500
H	0.07007500	0.11528700	1.91814900
Al	-1.26697200	0.02394400	-0.17662400
F	-0.69448700	-0.21386500	-1.72198000
F	-1.74787700	1.56393700	0.24580600
F	-2.00714300	-1.26535000	0.58119200

MDA-$AlCl_3$-c

C	-1.46735200	-1.20079900	-0.87983600
O	-2.46113400	-1.35208000	-0.07004100
H	-2.74816900	-0.41429300	0.19280600
C	-0.92758300	0.04035500	-1.20208500
C	-1.67311700	1.22857600	-0.79367400
H	-1.35333200	2.19593900	-1.19374000

O	-2.63288300	1.16182300	-0.01751200
H	-1.05576300	-2.12361000	-1.27497600
H	-0.25532400	0.09520300	-2.05073100
Al	0.86489700	0.01331900	0.18172800
Cl	1.90816400	-1.66026200	-0.59028500
Cl	1.61897800	1.92694500	-0.31570700
Cl	-0.03717900	-0.19686600	2.07764800

MDA-$AlBr_3$-c

C	1.47806800	-1.22765300	1.55283900
O	2.66358800	-1.40975800	1.07743800
H	3.03359100	-0.48110200	0.89639100
C	0.89132100	0.02856900	1.67837000
C	1.75197400	1.19613700	1.50013800
H	1.34859000	2.17448000	1.77916500
O	2.89708900	1.09880800	1.04496500
H	0.94805900	-2.13488200	1.82286500
H	0.00098700	0.11014400	2.29183100
Al	-0.41306500	0.01040300	-0.16561100
Br	-1.75983700	-1.75621900	0.30736200
Br	-1.31263800	2.07777100	0.09373500
Br	1.09604700	-0.24436900	-1.82980000

BH_2-MDA-BH_3-a

C	-0.36612900	-0.79449600	-0.00000700
O	-1.26309800	0.08451100	0.00024400
H	-0.35417300	1.52091400	-0.00000300
C	1.02700700	-0.46580500	0.00003400
C	1.38706100	0.87256900	-0.00001800
H	2.43109500	1.16612900	-0.00003500
O	0.56821600	1.88693800	-0.00006400
H	-0.68530500	-1.83804900	-0.00029300
B	2.11101400	-1.53751900	-0.00000400
H	1.81059800	-2.68817800	-0.00004800
H	3.25433700	-1.20670300	-0.00001700
B	-2.87184100	-0.31580700	-0.00010500
H	-3.26415600	0.19463000	1.01335800
H	-2.85316900	-1.52202100	-0.00018400
H	-3.26367100	0.19471300	-1.01372700

BH_2-MDA-BF_3-a

C	-0.84965900	0.69006300	0.00036400
O	0.03150700	-0.28171300	0.00071100
H	-0.52543700	-1.14453000	0.00057400

C	-2.21361700	0.47807100	-0.00006200
C	-2.64824500	-0.90500900	-0.00020100
H	-3.72704700	-1.10032400	-0.00075600
O	-1.86503400	-1.87241100	0.00020200
H	-0.41357300	1.68320400	0.00030900
B	-3.16937900	1.66021400	-0.00010800
H	-2.72924500	2.76684500	0.00011700
H	-4.34651400	1.47939200	-0.00022400
B	2.33543000	0.09885800	-0.00020900
F	2.12220100	1.40833100	0.00003500
F	2.54090700	-0.52774500	-1.14379300
F	2.54232500	-0.52788600	1.14305400

BH_2-MDA-AlH_3-a

C	0.07267600	-0.74052700	0.00084700
O	-0.78195000	0.18549700	0.00135200
H	0.21221600	1.55410100	0.00056700
C	1.47969400	-0.49604100	-0.00005000
C	1.91528400	0.82189700	-0.00033000
H	2.97401900	1.05796400	-0.00087700
O	1.15247600	1.87622600	-0.00008500
H	-0.29559900	-1.76933700	0.00091700
B	2.49746700	-1.63267900	-0.00041400
H	3.65856900	-1.37203100	-0.00105300
H	2.12595000	-2.76191400	-0.00005700
Al	-2.76617300	-0.14920300	-0.00057900
H	-3.15896800	0.57002400	1.36493100
H	-3.15511600	0.56718900	-1.36871900
H	-2.65829500	-1.74871800	0.00094400
